# The Concentration Determines Reaction Efficiency of Hetero‐Coupling of Bio‐Based Medium Chain Carboxylic Acids by Kolbe Electrolysis

**DOI:** 10.1002/cssc.70753

**Published:** 2026-05-25

**Authors:** Max Pohl, Katharina Röhring, Micjel Chávez Morejón, Falk Harnisch

**Affiliations:** ^1^ Department of Microbial Biotechnology UFZ – Helmholtz‐Centre for Environmental Research Leipzig Germany

**Keywords:** circular economy, electrobiorefinery, electrochemistry, green chemistry, power‐to‐fuels

## Abstract

Mixtures of seven *n‐*carboxylic acids (*n‐*CA), ranging from acetic acid to *n‐*octanoic acid, that can be gained from the microbial conversion of waste biomass, were converted into liquid drop‐in fuel via Kolbe electrolysis. We demonstrate a strong, sigmoidal correlation between the total *n*‐CA concentration and the Coulombic efficiency for fuel‐like liquid products (CE_fuel_). Crucially, only above an apparent threshold concentration of 0.1 mol L^−1^ *n‐*CA, Kolbe electrolysis takes place, whereas at lower concentrations the oxygen evolution reaction (OER) dominates. Optimal performance was achieved at the highest concentration tested, 1.5 mol L^−1^, yielding a maximum CE_fuel_ of 63.8 ± 1.4%, whereas for *n‐*CA to 0.1 mol L^−1^ and 0.5 mol L^−1^, the chain shortening reaction (CSR) strongly affects CE_fuel_. Furthermore, the study provides valuable insights into reaction selectivity, demonstrating that longer‐chain *n*‐CA react preferentially compared to shorter‐chain *n*‐CA, a phenomenon that is attributed to their higher hydrophobicity and hence accumulation in the hydrophobic layer formed on the electrode. Using a one‐chamber cell system at optimized concentration significantly lowered energy demand to 2.92 ± 0.04 kWhL^−1^ of fuel produced, representing a 40% reduction compared to similar two‐chamber setups.

## Graphical abstract

Total *n*‐carboxylic acid concentration governs the performance of Kolbe electrolysis for aqueous *n*‐carboxylic acid mixtures. Below an apparent threshold concentration of ~0.1 mol L^−1^^,^ only oxygen evolution takes place. Above this threshold, Kolbe electrolysis becomes dominant, reaching ~64% Coulombic efficiency for fuel‐like liquid products at 1.5 mol L^−1^.

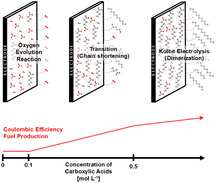

## 1 Introduction

Establishing a bio‐based circular economy is a major societal challenge. To achieve this goal, it is paramount to interweave the chemical and fuel sectors with renewable electricity generation and storage. One approach is electrification, which involves directly replacing fossil energy sources with renewable electricity. For example, in the transportation sector, electrification includes using green electricity in battery‐electric vehicles instead of gasoline, and in heating, it means using electric heat pumps instead of gas heaters [1]. However, replacing chemical energy vectors by direct electrification is often not feasible, for example, in heavy transportation or aviation [2]. Therefore, electrochemistry—and particularly electroorganic synthesis—holds great promise. Electroorganic synthesis, including electrochemical CO_2_ reduction and electrochemical hydrogenation, can produce renewable liquid and gaseous energy carriers from electricity [3, 4, 5]. Thereby, the feedstock (i.e., educt or substrate) of the electroorganic synthesis needs to be bio‐based, and thus the entire process line forms an electrobiorefinery. In general, electrobiorefineries can exploit a wide range of renewable feedstocks, particularly waste streams, and convert them into a broad spectrum of sustainable products [6].

One interesting concept of an electrobiorefinery that is aiming for energy carriers, in this case, liquid drop‐in fuels by converting bio‐based waste streams, was first introduced by Levy et al. [7] in the 1980s. In the first biological stage of the process, bio‐based waste streams are converted via anaerobic fermentation into mixtures of unbranched carboxylic acids (*n‐*CA). These *n‐*CA mixtures are then extracted and upgraded in the electrochemical stage via Kolbe electrolysis, which can be implemented in aqueous solution to generate fuel‐like liquid products, that are alkanes (Kolbe products) as well as oxygenates such as alcohols and esters (non‐Kolbe products), via different reaction pathways (see Figure S1). This electrobiorefinery was revived by Urban et al. [8], showing a maximum total carbon efficiency (expressed in chemical oxygen demand equivalents) of 0.5 g_fuel like liquid products_ $g_{\mathrm{corn}\mathrm{beer}}^{‐1}$ for the conversion of the real waste corn‐beer that is derived from ethanol production to liquid drop‐in fuel.

The biological stage has already been developed to a pre‐industrial scale, with demonstration [9] and pilot plants [10]. In contrast, the electrochemical stage is still being investigated systematically at laboratory scale and engineered towards validation in industrial environment. Significant progress has been made in understanding the mechanistic details [11, 12, 13, 14] of Kolbe electrolysis of *n*‐CA in aqueous solution and optimizing several reaction parameters and conditions such as pH, supporting electrolyte, and electrode material [11, 15, 16, 17, 18, 19]. However, almost all available studies have used only a single *n*‐CA with a chain length of two to eight carbon atoms (C_2_–C_8_) in an aqueous solution. Notably, in an electrobiorefinery using Kolbe electrolysis *n*‐CA mixtures in aqueous solutions, as gained from the bioprocess, need to be converted [9]. We recently narrowed this gap to application using mixtures of three *n*‐CA demonstrating that Kolbe electrolysis thereof shows increased Coulombic efficiency (CE) by hetero‐coupling when compared to Kolbe electrolysis of the single acids [15]. This study aims to further close this gap by studying the resemblance of an exemplary, bio‐based extraction solution. Specifically, it resembles the *n‐*CA‐enriched oil from the downstream processing (DSP) cascade of corn silage‐based fermentation broth, as shown in Braune et al. [20] extracted via liquid–liquid extraction with an alkaline aqueous solution (0.35 mol L^−1^ Na_2_CO_3_). The solution used for Kolbe electrolysis in this study is composed of a mixture of seven different *n*‐CA ranging from C_2_ to C_8_ in different proportions and containing salts (Na_2_CO_3_), being typical for DSP (see Table S1). This composition of the *n*‐CA mixture is comparable to extractants from similar waste‐utilizing bioprocesses, for example Carvajal‐Arroyo et al. [9].

Understanding how individual reaction parameters impact overall Kolbe electrolysis performance is crucial for optimizing the electrobiorefinery. In particular, the concentration of the individual *n*‐CA in the *n‐*CA mixture converted by Kolbe electrolysis is an essential process parameter, due to its immediate influence on the necessary extraction of *n‐*CA from the fermentation process, as well as on related parameters such as reaction time, which in turn affect the reactor and process design of the individual stages. Until now, the general assumption of Schäfer et al. [21] that a higher *n‐*CA concentration promotes Kolbe product formation has been widely accepted, but was surprisingly not systematically studied. Therefore, this study demonstrates that CE, yield (Y), and selectivity (S) (details on calculation shown below) of Kolbe electrolysis of *n*‐CA mixtures depend heavily on educt concentration, leading to improved conditions for producing fuel‐like liquid products at high concentrations. We also show that using a one‐chamber cell instead of a two‐chamber cell significantly lowers the energy demand in this specific setup.

## 2 Results and Discussion

### 2.1 Kolbe Electrolysis of a Seven‐Component n‐CA Mixture

The *n‐*CA mixture resembling the extraction solution from a bioprocess was used for Kolbe electrolysis at a total concentration of 0.5 mol L^−1^, considering all seven *n*‐CA (Table S1). Electrolysis was performed using platinized titanium as current state‐of‐the‐art electrode [19] for Kolbe electrolysis for 0.5 Faraday equivalents (FE) at conditions comparable to our previous studies [15, 19] to allow systematic comparison. The Kolbe electrolysis of the *n*‐CA mixture yielded over 150 products (see Table S3), necessitating extensive analytics as detailed in the experimental section. The targeted compounds were fuel‐like liquid compounds (Kolbe product: long‐chain *n‐*alkanes; non‐Kolbe products: short‐chain liquid hydrocarbons, alcohols, aldehydes, ketones, and esters; see Figure S1) [15, 19]. The obtained $S_{\text{fuel}}$ of 86.7 ± 10.6% was on par with or even higher than in previous studies on Kolbe electrolysis of *n*‐CA mixtures using only three acids (C_4_, C_6_, C_8_) (Neubert et al. [15], in the following denominated N1) and even single acids (C_6_) (Neubert et al. [19], in the following: N2)^1^ (see Table 1). The here achieved lower CE_fuel_ of 32.2 ± 6.0% can be explained by differences in experimental parameters such as using one‐ instead of two‐chamber cells, pH, ratio of electrode surface to solution volume, and especially the *n*‐CA mixture composition and used supporting electrolyte.

**TABLE 1:** Yield ($Y_{\text{fuel}}$), selectivity ($S_{\text{fuel}}$), and Coulombic efficiency (CE_fuel_) for the sum of fuel‐like liquid products in the organic phase per converted *n‐*CA for a *n‐*CA mixture in this study and Neubert et al [15]. (N1) as well as yield ($Y_{\text{decane}}$), selectivity ($S_{\text{decane}}$) and Coulombic efficiency (CE_decane_) for the production of the Kolbe product (decane) per converted *n*‐CA for C_6_ from Neubert et al. [19] (N2). Carbon balance and CE_overall_ take all Kolbe and non‐Kolbe products (liquid and gaseous, no OER) into account for all studies. The main differences of the three studies are the supporting electrolyte (this study: Na_2_CO_3_; N1/N2: Na_2_SO_4_), the composition of educt *n‐*CA (this study: C_2_–C_8_, N1: C_4_,C_6_,C_8;_ N2: C_6_) and the configuration of the reactor (this study: one‐chamber cell; N1/N2: two‐chamber cell). *n* provides number of replicates and ± represents the 95% confidence interval.

Study	Carbon balance, %	$Y_{\text{fuel}}$ or $Y_{\text{decane}}$, %	$S_{\text{fuel}}$ or $S_{\text{decane}}$, %	CE_fuel_ or CE_decane_, %	CE_overall,_ %
This study (*n* = 5)	44.2 ± 9.4	47.3 ± 10.3	86.7 ± 10.6	32.2 ± 6.0	35.3 ± 7.9
Neubert et al*.* [15] (N1) (*n* = 3)	86.1 ± 3.7	80.1 ± 4.7	78.5 ± 1.9	67.5 ± 2.2	80.3 ± 1.2
Neubert et al*.* [19] (N2) (*n* = 6)	65.0 ± 7.5	52.3 ± 6.9	66.9 ± 0.9	48.3 ± 3.2	69.2 ± 3.4

Decreased performance was expected, since we moved towards realistic process conditions by mimicking a bio‐based extraction solution. Among these more realistic process conditions, Na_2_CO_3_ was used as supporting electrolyte, which is required for the extraction of *n*‐CA from the bioprocess, while previously Na_2_SO_4_ was used (N1/N2). Notably, the molar concentration of the supporting electrolyte (this work: 0.35 mol L^−1^, N1/N2: 0.25 mol L^−1^) as well as the electrolytic conductivity (here: 50.8 ± 1.3 mS cm^−1^; N1: 47.1 ± 0.7 mS cm^−1^; N2: 42.3 ± 0.4 mS cm^−1^) were similar. As reported by Stang et al. [17], changing the supporting electrolyte may significantly impact the CE by affecting the reaction selectivity. Specifically, Nordkamp et al*.* [16] and Baumgarten et al. [22] reported the negative impact of carbonate species on Kolbe electrolysis through interactions at the electrode–electrolyte interface, postulating that HCO_3_^−^ ions play a decisive role. Due to the slightly alkaline pH of around 9 in this study (pH = 8.2 ± 0.3 (N1) and pH = 7 (N2)) and the high carbonate content, based on the carbonate equilibrium (see Figure S3), the HCO_3_^−^ ion concentration is high. This enhances the likely strong negative effect of the carbonate species on the Kolbe electrolysis due to the impaired formation of a sufficient hydrophobic layer needed for decarboxylation and stabilizing effects of formed intermediates that support side reactions [16, 22].

The highly complex composition of the *n‐*CA mixture is certainly the most important difference compared to all previous studies. Using a greater variety of *n‐*CA leads to a significantly broader product spectrum due to an exponentially increased number of possible reaction pathways, especially through hetero‐coupling reactions of the radicals formed from *n‐*CA (see Table S3). This challenges qualitative and quantitative analysis of the product mixture that may lead to an underestimation of the products formed, particularly for compounds quantified via class‐average response factors. This likely contributes to the low carbon balance of about 50% (see Table 1). Additional contributions include systematic under‐accounting of inorganic carbon (CO_2_/HCO_3_^−^/CO_3_^2−^) in alkaline solutions due to the absence of outgassing by post‐electrolysis acidification.

Further, using a one‐chamber cell in this study, as opposed to the two‐chamber cells used in N1 and N2, likely negatively impacts the performance. As shown by Neubert et al. [19], for Kolbe electrolysis when using only C_6_, the CE_decane_, that is the Kolbe product, was approximately 15% lower when using a one‐chamber cell compared to a two‐chamber cell. Despite this, from an energetic point of view using a one‐chamber cell is strongly preferred as the electric energy demand is significantly lower per mole of educt converted for similar reaction conditions as a consequence of the lowered internal resistance compared to those systems (this study: 303.4 ± 6.1 Wh mol^−1^; N1: 402.6 ± 12.3 Wh mol^−1^), while due to the different CE_fuel_ the energetic advantage of the one‐chamber cell for the fuel production is partly leveled off at this concentration (this study: 1.16 ± 0.02 kWh mol^−1^; N1: 0.98 ± 0.06 kWh mol^−1^).

### 2.2 The Influence of n‐CA Concentration on Kolbe Electrolysis

To optimize the extraction of *n‐*CA from the biological process for use as a substrate for Kolbe electrolysis, the effect of *n‐*CA concentration on yield ($Y_{\text{fuel}}$), selectivity ($S_{\text{fuel}}$), and Coulombic efficiency (CE_fuel_) was investigated. Therefore, nine different concentrations of the *n‐*CA mixture ranging from 0.0 mol L^−1^ to 1.5 mol L^−1^ using identical *n‐*CA ratios were studied (see Tables S1 and S6).

**FIGURE 1:**
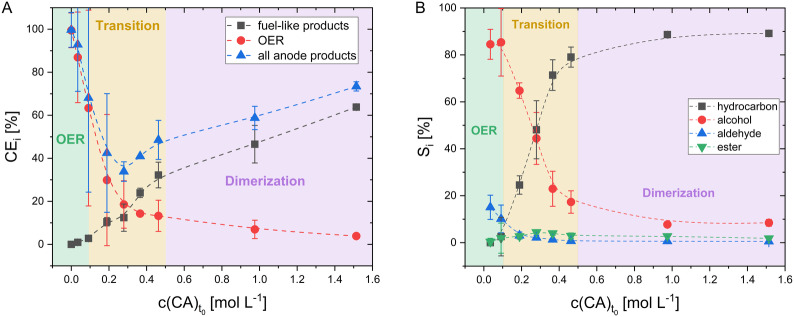
Kolbe electrolysis of *n*‐CA mixtures containing C_2_ to C_8_ with different initial *n‐*CA concentrations ($c(\mathrm{CA})t_{0}$) ranging from 0 to 1.5 mol L^−1^ in a one‐chamber reactor with 150 mA cm^−2^ up to 0.5 FE at pH = 9. (A) CE_*i*_ of fuel‐like liquid products (CE_fuel_), the OER (CE_OER_) and the total products formed at the anode (CE_anode_) for the different $c{\left(\mathrm{CA}\right)}_{t0}$ are presented. (B) Selectivity $S_{i}$ relative to the fuel‐like liquid products of hydrocarbons, alcohols, aldehydes and esters for the different $c{\left(\mathrm{CA}\right)}_{t0}$. The colored areas indicate the dominant process in the corresponding concentration range. Data points are connected with a dashed line to guide the eye (no interpolation). The values shown are averages of the replicates (at 0.5 mol L^−1^: *n *= 5; at all other concentrations: *n* = 3) and the error bars represent the 95% confidence interval. Please note that small error bars are hidden by the symbol of the data point.

Figure 1A shows the CE of three different processes—namely, CE_fuel_ of fuel‐like liquid products, the CE_OER_ of the oxygen evolution reaction (OER), and CE_anode_, considering all anodic products as a function of the initial *n‐*CA concentrations. Clearly, an increasing initial *n‐*CA concentration leads to an increase in CE_fuel_ and hence decline in CE_OER_. The counter reaction at the cathode, the hydrogen evolution reaction (HER), shows a constant ${\mathrm{CE}}_{H_{2}}\geq 80\%$, independent of the initial *n‐*CA concentration, strongly suggesting the gas analytical methods are reliable and hence support the clear impact of the initial *n‐*CA concentration on CE_fuel_.

The plot of CE_fuel_ versus *n‐*CA concentration is sigmoidal and can be divided into three areas (see Figure 1A). First, in the OER area, that is at low *n‐*CA concentrations of up to 0.1 mol L^−1^, Kolbe electrolysis toward fuel‐like liquid products barely occurs (CE_fuel_ < 3%), and the OER is dominant (represented by CE_OER_). In the electrolysis of aqueous solutions, the OER is typically the main reaction at the anode [23]. However, in the presence of carboxylates, a hydrophobic layer forms on the electrode surface. This layer (partially) suppresses the OER and enables Kolbe electrolysis (compare Figure S5) [21]. As demonstrated in Figure 1 (A), this effect is concentration‐dependent, with hyperbolic decline of CE_OER_ from 99.5 ± 8.1% at 0 mol L^−1^ to ~60% at 0.1 mol L^−1^ and below 5% at 1.5 mol L^−1^. This phenomenon can be explained by the increasing uniformity and thickness of the hydrophobic layer on the anode surface with increasing *n‐*CA concentration. However, the OER is not fully suppressed, even at concentrations above 0.5 mol L^−1^ (see Table S6), unlike what is shown by Neubert et al. [15], where CE_OER_ for Kolbe electrolysis of 0.5 mol L^−1^ *n*‐CA mixtures was reduced below 1% when using Na_2_SO_4_ as supporting electrolyte. This unexpected behavior can be explained by the above‐discussed findings of Baumgarten et al. [22] showing that high HCO_3_^−^ ion concentration, as present here due to the usage of Na_2_CO_3_ as supporting electrolyte at pH = 9, leads to an insufficient hydrophobic layer, enhancing OER.

Second, in the transition area, that is for *n‐*CA between 0.1 mol L^−1^ and 0.5 mol L^−1^, CE_fuel_ increases steeply due to concentration‐dependent parameters favoring the Kolbe electrolysis. Third, in the dimerization area that is for *n‐*CA above 0.5 mol L^−1^, the CE_fuel_ increases further gradually from 32.2 ± 6.0% to 63.8 ± 1.4% while CE_OER_ declines to < 15%. The steady, but less steep, increase of CE_fuel_ with increasing *n‐*CA is due to the rising impact of concentration‐dependent processes that may limit reaction kinetics of Kolbe electrolysis, such as limited product desorption from the electrode, current density limitations, and solubility effects, such as micelle formation or local insolubility due to local pH changes. The effect of those mass transport limitations, potentially caused by the applied current or stirring speed, does need further examination. We speculate that it might even result in a changed concentration range of the dimerization area, which opens new avenues for reactor and reaction engineering.

As shown in Figure 1 B, the selectivity of fuel‐like liquid products toward hydrocarbons increases to over 80% in this area. The increase in liquid hydrocarbons and the tendency toward favorable reactions is also represented in $Y_{\text{fuel}}$ (see Figure S4), leading to an increasing share of products that we could detect, finally reaching 83.3 ± 16.5% at 1.5 mol L^−1^. At this concentration, a maximum CE_fuel_ of 63.8 ± 1.4% is reached, which is close to the conversion of a less complex mixture containing only C_4_, C_6_, and C_8_ under optimized conditions as reported earlier (67.5 ± 2.2%, see Table 1). A further increase in the initial *n‐*CA concentration may result in an even higher CE_fuel_, this can also be limited by the solubility of the individual *n*‐CA.

The shift towards CE_fuel_, especially the increase of CE_liquid hydrocarbons_, with an increased initial *n‐*CA concentration is primarily due to the suppression of OER and likely the chain‐shortening reactions (CSR; see Figure S1), finally favoring Kolbe product formation by dimerization. The formation of non‐Kolbe products is low and independent of concentration, as shown by a constant CE_non‐Kolbe_ between 5% and 8% (see Table S6). The suppression of the OER and CSR can be mechanistically explained by the increasing *n‐*CA and hence carboxylate concentrations in the solution and especially at the electrode surface [24]. This leads to an increased radical concentration on the electrode surface, lowered spatial spacing between radicals formed by decarboxylation and a changing ion ratio of carboxylates to other anions (carbonate species and OH^‐^). These conditions result in a higher radical recombination probability that is dimerization, thereby increasing the share of Kolbe products, as already assumed by Schäfer et al. [21]. Furthermore, it can be speculated that, due to the increasing carboxylate concentration and changed ion ratio, the hydrophobic layer formed by the carboxylates on the electrode becomes thicker and more uniform. Thus, investigating the hydrophobic layer further would be beneficial for a deeper mechanistic understanding. For example, by further examination of the surface coverage of the electrode via electrochemical impedance spectroscopy, as demonstrated by Ashraf et al. [11].

#### 2.2.1 Threshold Concentration of the Kolbe Electrolysis

In the OER area up to 0.1 mol L^−1^ *n‐*CA, nearly no Kolbe products are formed (CE_fuel _< 3%, see Table S6; *S*_hydrocarbon _<  3% Figure 1B), as also supported by the lack of acid degradation (see Table S6). This leads to the conclusion that there is a threshold concentration of *n‐*CA below which the Kolbe electrolysis does not occur. This is most likely due to insufficient formation of a hydrophobic layer at the surface and lower onset potential of the OER when compared to decarboxylation of the *n*‐CA. This is supported by Nilges et al. [25] showing the lower onset potential that is effect of the hydrophobic layer, as seen when comparing cyclic voltammograms of the oxidation of valeric acid in aqueous electrolyte solution (Kolbe electrolysis) and the pure electrolyte solution (OER).

This finding is strongly supported by experiments with prolonged Kolbe electrolysis at 2.5 FE for multiple initial *n‐*CA concentrations (0.2 mol L^−1^ to 1.0 mol L^−1^). An end *n‐*CA concentration of 0.10 ± 0.03 mol L^−1^ was reached in all experiments, independent of the initial *n‐*CA concentration. This result contradicts the often implicit assumption that the significant electron excess would lead to complete *n‐*CA conversion (see Figure S6). Specifically, under the investigated conditions an apparent threshold concentration for Kolbe electrolysis of 0.1 mol L^−1^ is present. The dependency of this principal phenomenon from different parameters such as surface area to volume ratios, varying mass transport regimes, electrolyte type and concentration, pH or current density needs to be investigated further. One may speculate that the composition of the *n*‐CA mixture also has a strong impact and that an increasing share of longer‐chain *n*‐CA leads to a lowered threshold concentration due to a higher hydrophobicity of the hydrophobic layer on the electrode surface hindering the OER.

#### 2.2.2 The Impact of the Chain‐Shortening Reaction (CSR)

It could be expected that CE_anode_, which represents the electron efficiency for all products formed at the anode (Kolbe and non‐Kolbe products, O_2_), is constant and independent of the initial *n‐*CA concentration. However, CE_anode_ forms a local minimum in the transition area (see Figure 1A). The local minimum in CE_anode_ is most likely caused by a higher electron demand per product than assumed in the calculations. This occurs, if a fraction of intermediates undergoes CSR prior to forming the detected products, increasing the effective electron demand per mole of product. In CSR, the *n‐*CA chain is shortened by one C atom as a consequence of the formation of a non‐Kolbe‐product that is first an alcohol, and subsequent oxidation to *n‐*CA via an aldehyde. This prolonged reaction pathway requires at least additional 6 e^−^. For instance, dimerization of two *n‐*CA molecules each undergoing a CSR of one C atom would increase the electron demand from 2 e^−^ to up to 14 e^−^ (see Figure S1) for the Kolbe product. The possible, massive impact of CSR, which is likely to happen in the transition area, is illustrated by an example for the reaction at an initial *n‐*CA concentration of 0.5 mol L^−1^. When assuming that only 13.8 ± 2.4% of the reacted *n‐*CA would have undergone a CSR by one C atom, the gap between 100% and experimentally determined CE_anode_ of 48.5 ± 9.1% would be closed.

The occurrence of the CSR cannot be directly proven in this set of experiments as an extraordinary precise quantification of CO_2_ (the byproduct of CSR) would be required and in mixtures containing all *n‐*CA from C_2_ to C_8_, there are no specific CSR products.

Noteworthy, our findings are in line with literature providing several hints for the occurrence and impact of the CSR. For example, Neubert et al. [15] demonstrated the detection of heptanoate (a CSR product) during Kolbe electrolysis of C_8_. One indicator of the CSR can be found by analyzing the specific degradation of individual *n‐*CA. As discussed in detail below, *n‐*CA degradation decreases with decreasing chain length. At low concentrations (transition area) and with an increased reaction duration (2.5 FE), the formation instead of degradation of short‐chain *n‐*CA (C_2_ and C_3_) was detected (see Figure S7) for which CSR is the only conceivable reaction pathway. Another indicator can be seen in Figure 1B, which shows that selectivity toward formation of alcohols, and especially aldehydes (intermediates of the CSR; see Figure S1), is much higher than toward hydrocarbons at low concentrations compared to higher concentrations in the transition area.

The occurrence of the CSR at low concentrations may be explained by the reduced radical density on the electrode surface, which results in lower coupling probabilities for dimerization and a higher probability of further electrochemical oxidation of radicals to alcohols, aldehydes and shorter‐chain *n‐*CA. Additionally, the high carbonate concentration at the electrode surface may favor further radical oxidation through stabilizing effects on CSR intermediates on the electrode surface. Baumgarten et al. [22] observed for Kolbe electrolysis of C_8_ this phenomenon through an increasing formation of aldehydes as a CSR intermediate with increasing carbonate concentrations (further oxidized *n‐*CA was not analyzed or reported). To gain deeper insight into the effects of CSR and quantify its impact, experiments focusing on CSR with single *n‐*CA or *n‐*CA mixtures containing isotopically labeled *n‐*CA are necessary.

#### 2.2.3 Effect of *n*‐CA Chain Length on the Reactivity

**FIGURE 2:**
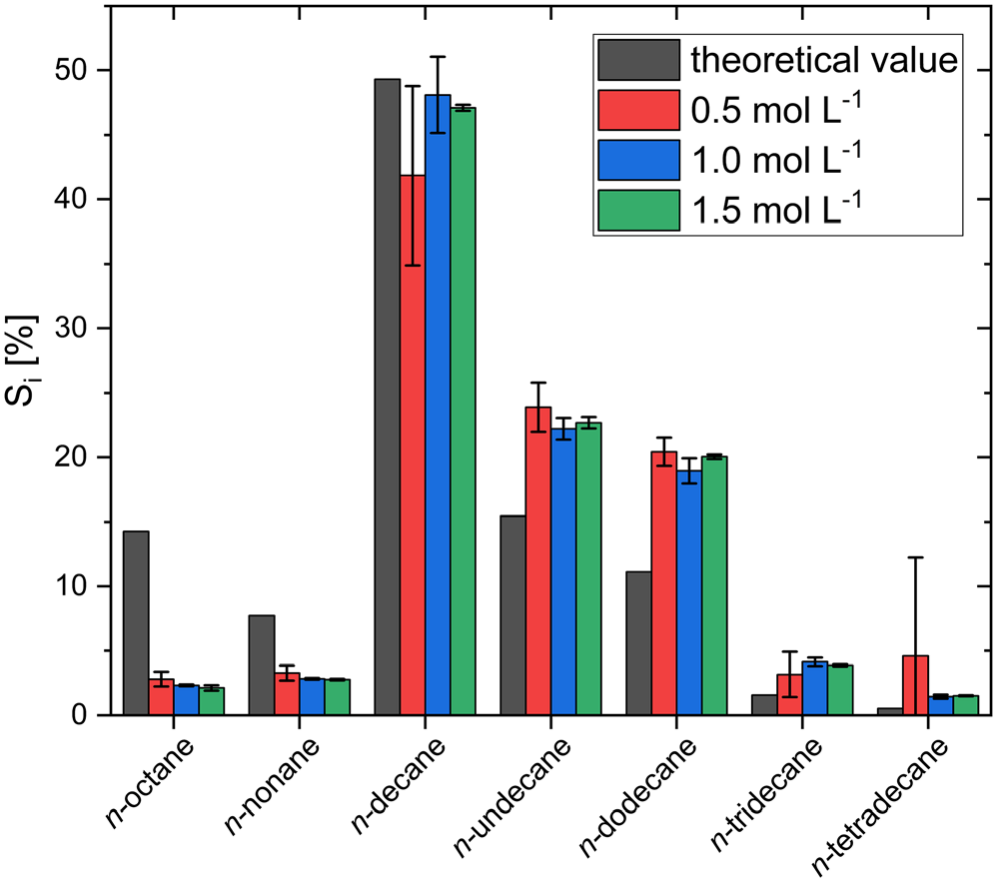
Normalized selectivity *S*_norm, *i*_ toward liquid dimerization products (*n*‐alkanes: *n‐*octane to *n*‐tetradecane). The shown values are measured for different initial *n‐*CA concentrations from 0.5 mol L^−1^ to 1.5 mol L^−1^ and the theoretical values *S*_theo,norm,*i*_ are calculated based on the distribution of the individual *n‐*CA in the *n‐*CA mixture (for detailed calculation: see Table S7). The values shown are averages of the replicates (*n* = 3, at 0.5 mol L^−1^: *n* = 5) and the error bars represent the 95% confidence interval.

The composition of the fuel‐like liquid products formed during Kolbe electrolysis is of special interest for its usage in further application, such as kerosene or diesel. The composition is determined by the reaction behavior of the individual *n‐*CA in the *n‐*CA mixture. It can be assumed that the distribution of *n‐*alkanes being derived from dimerization depends on the proportion of individual *n‐*CA in the mixture. This distribution can be approximated by assuming that the formation and coupling probabilities of all radicals are identical for all *n‐*CA and that the encounter probabilities depend on the proportions of the individual *n‐*CA in the mixture (see Table S7). Based on these assumptions, theoretical selectivities $S_{\text{theoretical}}$ were calculated for the different dimerization products. By comparing $S_{\text{theoretical}}$ with the experimental selectivities of the dimerization area, a different trend emerges (see Figure 2). The experimental data shows that the formation of longer‐chain *n*‐alkanes (undecane to tetradecane) from the dimerization of longer‐chain *n‐*CA (C_6_–C_8_) is strongly preferred when compared to their theoretical values. In contrast, the formation of shorter‐chain *n*‐alkanes (octane and nonane) from the dimerization of shorter‐chain *n‐*CA (C_4_/C_5_ and C_6_) is less preferred. This behavior is consistent across all examined initial *n‐*CA concentrations. The individual acid degradation rates (see Figure S7) underline the trend that longer‐chain *n‐*CA react preferentially compared to shorter‐chain *n‐*CA.

A possible explanation for the deviation is that the calculation of $S_{\text{theoretical}}$ assumes that the hydrophobic layer formed by the carboxylates on the electrode surface is composed based on the concentration of the individual *n‐*CA in the bulk liquid. This is most likely not the case as the real composition will also be determined by the hydrophobicity of the layer formed by carboxylates and hydrophobic products itself. Thus, we hypothesize that longer‐chain *n‐*CA with a higher hydrophobicity, represented by a higher *n*‐octanol/water partition coefficient P (e.g., log *P*(*C*_8_) = 3.05), may be enriched in the interfacial hydrophobic layer than shorter‐chain *n‐*CA with a low P (e.g., log *P*(*C*_2_) = −0.17) [26]. This composition of the hydrophobic layer directly influences the probability of radical formation, their reaction behavior and hence, the product distribution. In summary, longer‐chain *n‐*CA are more likely to react than shorter‐chain *n‐*CA in *n‐*CA mixtures.

These findings are consistent with previous research. Schäfer et al. [21] proposed that different preferential adsorption on the electrode, as described here, caused by the hydrophobic layer, might favor certain *n‐*CA over others. Levy et al. [27] further support this hypothesis by showing that the hydrophobic layer in mixtures of C_6_ with C_3_ or C_4_ mainly consists of the longer‐chain C_6_, which also reacts preferentially. However, this effect probably depends on the mass transport regime and the formation of the hydrophobic layer. A similar behavior was not detected by Ziogas et al. [28], probably due to the use of a microflow reactor, which affected the formation and especially thickness of the hydrophobic layer by sheer stress.

### 2.3 Consequences for Implementation

As shown, a concentration of *n‐*CA above the threshold concentration of at least 0.1 mol L^−1^ is necessary to partially suppress the OER and enable Kolbe electrolysis. To further improve the efficiency for forming fuel‐like liquid products, the concentration should be in the dimerization area that is above 0.5 mol L^−1^, where a higher concentration leads to a higher CE_fuel_. This knowledge is highly important for developing and tuning the extraction process in the electrobiorefinery to the optimal concentration—as high as possible under efficient conditions. Therefore, a continuous resaturation of the reaction solution with *n*‐CA by constant extraction via membrane‐based liquid–liquid extraction or a semi‐continuous approach using two separate reaction solution cycles, in which saturation and electrolysis happen in parallel, could be feasible technical approaches to run the Kolbe electrolysis under optimal conditions that need to be proven by longer time operation in future.

In addition to improving efficiency by increasing the *n‐*CA concentration, implementing a one‐chamber system instead of a two‐chamber system significantly lowers the electricity costs for electrolysis for the here compared systems, even if the CE_fuel_ is in the same range. In this simplified scenario, the electricity cost per liter of fuel decreases by 40%, from 5.02 kWh L^−1^ (Neubert et al. [15]) to 2.92 ± 0.04 kWh L^−1^ (1.5 mol L^−1^), resulting in a fuel price of 52.0 ± 0.7 ct L^−1^ (see Table S8). The main reason for this reduction is the significantly lower internal resistance due to no need for a membrane. Nevertheless, a detailed assessment of the setup and operation of the electrochemical reactor including a techno‐economic assessment (compare to Matthiesen et al. [29]), is necessary. Considering the negative effects on Kolbe electrolysis of changing the supporting electrolyte from Na_2_SO_4_ to Na_2_CO_3_, which was necessary to mimic the extraction, further optimization and cost savings appear feasible, e.g., by choosing a different extraction method. From a process point of view, it is also highly relevant that longer‐chain *n‐*CA are more likely to react than shorter‐chain *n‐*CA in *n‐*CA mixtures (at least in batch systems). This effect is beneficial when aiming for fuel such as kerosene, consisting mainly of dimers of long‐chain *n*‐CA, and must be kept in mind when tuning the composition of the bio‐based *n‐*CA mixture towards the optimal product composition after Kolbe electrolysis.

## 3 Conclusion

The investigation of a seven‐component *n*‐CA mixture shows a clear correlation between increasing total *n*‐CA concentration and improved Kolbe electrolysis performance, represented as CE_fuel_. The maximum CE_fuel_ of up to 63.8 ± 1.4% was achieved at the maximum *n‐*CA concentration of 1.5 mol L^−1^. The first successful in‐depth analysis of a complex *n‐*CA mixture containing seven different *n‐*CA used in Kolbe electrolysis revealed an observed preference for long‐chain *n‐*CA over shorter‐chain *n‐*CA in *n‐*CA mixtures. Concentration‐dependent effects, such as CSR for medium concentrations, were postulated, and an apparent threshold concentration of ~0.1 mol L^−1^ was observed under the present conditions. The transferability of this threshold concentration to other systems with different surface area to volume ratios or varying mass transport regimes [30] needs to be examined. Further insights into the behavior of the hydrophobic layer and the effects of foreign ions could lead to a better mechanistic understanding of these effects and guide process engineering. Adjusting the reactor to one‐chamber design and taking advantage of the concentration effects resulted in significant energy savings of up to 40% for fuel production via Kolbe electrolysis compared to our previous study [15].

These improvements and the deepened knowledge of Kolbe electrolysis of complex *n*‐CA mixtures based on the extraction from the bioprocess brings the electrobiorefinery process one step closer to application. The next steps in developing the electrobiorefinery for fuel from waste production include further research on Kolbe electrolysis, especially for use of curtailed electric power, conversion of increasingly complex solutions of CA, developing a scalable reactor depending on the extraction stage, and process intensification by integrating the biological stage with the electrochemical stage via the extraction stage, especially for long‐term operation.

## 4 Experimental Section

### 4.1 General Remarks

All chemicals were of at least analytical grade. All solutions were prepared with deionized water (Milli‐Q IQ 7000, Merck KgaA, Darmstadt, Germany). A list of all used symbols and abbreviations, as well as details of chemicals and applied analytical methods, can be found in the Supporting Information.

### 4.2 Experimental Setup

Electrochemical conversion of *n‐*carboxylic acid mixtures (*n‐*CA with C_2_–C_8_) by Kolbe electrolysis was performed in a 100 mL reactor (Schott AG, Mainz, Germany) in one‐chamber configuration with a working volume of 50 mL, as described in detail previously (see Figure S2) [19]. Briefly, the reactor was equipped with the working electrode (WE), the counter electrode (CE, 18 mm distance to WE), the reference electrode (RE), and two needle ports. Additionally, one replicate hosted a pH electrode. The whole setup was fabricated gas‐tight, except for the two needle ports for gas inlet and gas outlet.

All experiments were carried out galvanostatically using a DC power source (2230‐30−1 triple Channel DC Power Supply, Keithley/Tektronix GmbH, Köln, Germany) in a three‐electrode configuration consisting of platinized titanium WE and CE (Umicore, Schwaebisch Gmuend, Germany) with a geometric surface area of 2 cm^2^ and an Ag/AgCl sat. KCl reference electrode (SE 11, Xylem Analytics Germany Sales GmbH & Co. KG/Sensortechnik Meinsberg, Waldheim, Germany). The DC power supply was connected to the WE serving as anode and the CE serving as cathode. The cell potential (*E*_cell_) between WE and CE was measured using the DC power supply. The anode potential was measured relative to the RE for one replicate per set of experiments with an additional multimeter (Autoranging Mini MultiMeter MN16, Extech Instruments, Nashua, USA).

The reaction solution was purged with N_2_ for at least 10 min before each electrolysis. For all connections, gas‐tight Tygon tubes (Saint‐Gobain, Charny, France) were used. The pH, temperature, and conductivity were measured before and after the experiment. Additionally, continuous pH and temperature measurements were performed for one replicate per set of experiments. All measurements of pH, temperature, and conductivity were performed using a SevenExcellence S470 (Mettler‐Toledo, Greifensee, Switzerland) with an InLab Micro Pro pH electrode and an InLab 710 conductivity electrode (both Mettler‐Toledo, Greifensee, Switzerland). Both electrodes were calibrated with commercial buffer solutions (Mettler‐Toledo, Greifensee, Switzerland) directly before each experiment. After each electrolysis, the electrodes and all other inlet parts of the setup were cleaned with water.

### 4.3 Kolbe Electrolysis

To prepare the reaction solution, 75–105 mL of water were introduced into a 250 mL flask. Then, the corresponding amounts of seven different *n‐*CA were added to obtain 175 mL solution of the defined total concentration ranging from 0 to 1.5 mol L^−1^, as detailed in Table S1, resembling the product of a downstream processing cascade of corn silage‐based fermentation broth, Braune et al. [20]. Subsequently, the pH of the solution was adjusted to pH = 7 ± 0.3 using NaOH (pellets and/or 10 mol L^−1^ NaOH) and 3 mol L^−1^ H_2_SO_4_ to enable the addition of Na_2_CO_3_ without direct pH caused CO_2_ outgassing. Afterwards, 61 mL of 1 mol L^−1^ Na_2_CO_3_ was added to reach a final Na_2_CO_3_ concentration of 0.35 mol L^−1^. The pH was then adjusted to 9.0 ± 0.1 using 10 mol L^−1^ NaOH and 3 mol L^−1^ or 6 mol L^−1^ H_2_SO_4_. Afterwards, water was added up to 175 mL.

50 mL of the reaction solution was used for electrolysis, and 1 mL was taken as blank sample (rest remained as back‐up for further experiments). The 50 mL of the electrolysis solution was filled into the reactor and the reactor was weighed. Thereafter, the complete experimental setup was assembled. The gas outlet was connected to a N_2_‐mass flow meter (MFM; EL‐FLOW Prestige FG‐201CV (max. 50 mL_*n*_ min^−1^), Bronkhorst High‐Tech B.V., Ruurlo, Netherlands) in order to continuously determine the volume of produced gas during the experiment. A gas bag was used to collect the produced gases for measuring their composition after electrolysis with a microGC (Inficon Fusion Micro GC, Inficon, Köln, Germany). The electrolysis was carried out for 0.5 FE relative to the initial *n‐*CA concentration using a constant current of 300 mA at room temperature and 800 rpm stirring. After the electrolysis, the reactor was kept gas‐tight, and the reaction chamber was purged with at least 100 mL_*n*_ N_2_ controlled via the N_2_‐mass flow meter to quantify the remaining gas in the dead volume.

For long‐term experiments, the electrolysis was increased to 2.5 FE and online gas phase measurements were performed by connecting the gas outlet to the microGC in a by‐pass configuration. More details regarding the electrochemical parameters are provided in Table S2.

### 4.4 GC–MS for Quantification of *n*‐CA and Liquid Electrolysis Products

To quantify the amount of reaction products in the liquid, organic, and aqueous phases, the organic phase was extracted with 2 mL *n*‐hexane and the phases were separated in a separation funnel. Both phases were separately collected into flasks. The weights of the organic and aqueous phases were determined. The density of the phases and of the reaction solution were determined by weighing 1 mL.

For each experiment, a sample of 1 mL was taken from the reaction solution before the Kolbe electrolysis ($t_{0}$). The samples at *t*_0_ were diluted to two dilution levels between 1:5 and 1:50 or 1:60 and 1:600 with acidic water, depending on the initial *n‐*CA concentration. The sample of the aqueous phase after Kolbe electrolysis was diluted 1:10 and 1:100 with acidic water, except for highly concentrated samples for which higher dilutions were used. The samples of the organic phase were diluted to 1:10, 1:100, and 1:1000 with *n*‐hexane and 1:10 and 1:100 with dichloromethane (DCM). Undecanoic acid methyl ester was used as an internal standard for the samples of the organic phase and cyclohexanone as internal standard for the aqueous phase. The samples were analyzed by gas chromatography‐mass spectrometry (GC–MS) (GC 7890A and MSD 5975C InertXL; Agilent Technologies, Santa Clara, USA) using a DB‐FATWAX capillary column (30 m × 250 μm × 0.25 μm; Agilent Technologies, Santa Clara, USA) with He as carrier gas. The column temperature was held at 50°C for 2 min and then increased to 250°C using a 15 K min^−1^ ramp. *n‐*CA, *n*‐alkanes, alcohols and esters were identified based on the retention times and mass spectra of pure reference compounds. For the aqueous phase, *n*‐CA (C_2_−C_6_ and C_7_−C_8_, 3 levels each) and alcohols (C_2_−C_6_, 3 levels) were quantified using external standards. For the organic phase, *n*‐CA (C_4_‐C_10_, 4 levels), *n*‐alkanes (C_5_−C_7_ and C_8_−C_18_, 4 levels each), alcohols (C_3_−C_8_, 3 levels), and some esters (C_10_−C_11_, 3 levels) were quantified using external standards. Unknown compounds were identified using mass spectra and the NIST 17 mass spectra library. Compounds without external standards were quantified by an average response factor of all calibrated substances in the compound class. This was performed for all compounds with a relative peak height of at least 0.1%, an ion peak signal‐to‐noise ratio of above 0.1%, a sharpness of over 25%, and a library match factor of over 70%. A detailed overview of the quantified and calibrated compounds can be found in Table S3.

### 4.5 Gas Phase Analysis

The gas phase analysis was performed using a four‐channel microGC (Inficon Fusion Micro GC, Inficon, Köln, Germany) equipped with a thermal conductivity detector (TCD). A detailed description of the specification can be found in Table S4. The device was calibrated to analyze the experimental gas profile containing H_2_, O_2_, N_2_, CO_2_/CO, and various gaseous hydrocarbons (C_1_−C_6_). The single calibration levels, compounds and concentrations can be found in Table S5.

For all experiments, the total gas composition was analyzed using the gas collected in the gas bags (offline measurements). For the long‐term experiments, the composition of the gas phase during electrolysis was analyzed using a by‐pass configuration (online measurements). These measurements were carried out at the beginning, then every 10 min for the first 60 min, and every 20–30 min for the rest of the experiment, depending on the total length. The microGC‐TCD measurements resulted in the mole fraction $y_{i}$ of each individual gas component i in % and the gas volume $V_{\text{output}}^{\mathrm{norm}}$ in mL was obtained from the MFM.

### 4.6 Data Processing and Calculations

For quantification of individual components in gas mixtures, data from the MFM and the microGC‐TCD measurements were combined. This allowed to calculate the absolute amount of each gaseous component that was produced during electrolysis. All gas phase calculations are based on Neubert et al*.* [31].

The CE for each compound i, CE*_i_* was calculated via Equation (1) by relating the charge *Q* *_i_*, which was theoretically transferred to consume or produce the compound i, to the total charge $Q_{\text{total}}$, which was transferred during electrolysis:

(1) $${\mathrm{CE}}_{i}=\frac{Q_{i}}{Q_{\text{total}}}\times 100\%$$

The charge *Q* *_i_* was determined via Equation (2) using the amount of substance *n* *_i_*, the number of transferred electrons per molecule *z* *_i_*, and the Faraday constant F:

(2) $$Q_{i}=n_{i}\times z_{i}\times F$$

The number of electrons transferred *z* *_i_* for the different compounds depends on the assumed dominant reaction pathway (see Figure S1) and can be found in Table S3 (possible CSR pathways are not included in the stoichiometric electron accounting; therefore, CE values represent apparent efficiencies under the assumed dominant pathways, more details in section “The impact of the chain‐shortening reaction (CSR)*”*). In the case of *n‐*CA analyzed in the aqueous phase prior to electrolysis, the amount Δn represents the difference between the initial value ($t_{0}$) and the value after the electrolysis.

The total charge $Q_{\text{total}}$ was derived *via* Equation (3) by integrating the current I over the reaction time t:

(3) $$Q_{\text{total}}=\int I(t)dt$$

The Faraday equivalents FE were calculated by dividing the total charge $Q_{\text{total}}$ by the charge $Q_{n-\mathrm{CA},t_{0}}$ necessary to convert all *n‐*CA contained in the start solution (Equation (4)). The initial amount of *n‐*CA that is $n_{n-\mathrm{CA},t_{0}}$ was obtained by analyzing the solution by GC–MS before the reaction. The charge $Q_{n-\mathrm{CA},t_{0}}$ was calculated summing up *Q* *_i_* gained via Equation (2) for all educts.

(4) $$\mathrm{FE}=\frac{Q_{\text{total}}}{Q_{n-\mathrm{CA},t_{0}}}$$

The compound‐specific yield *Y* *_i_* was determined by dividing the amount of a specific compound *n* *_i_* multiplied by the number of *n‐*CA necessary to form this compound $x_{n-\mathrm{CA}}$ by the consumed amount of *n‐*CA $\Delta n_{n-\mathrm{CA}}$ (Equation (5)). The number of *n‐*CA molecules required for the shortest reaction pathway for formation of this compound $x_{n-\mathrm{CA}}$ (possible CSR are not considered here) is two for *n‐*alkanes formed by dimerization and esters, and one for all other compounds (see Table S3).

(5) $$Y_{i}=\frac{x_{n-\mathrm{CA}}\times n_{i}}{\Delta n_{n-\mathrm{CA}}}\times 100\%$$

The selectivity *S* *_i_* was calculated by Equation (6) when dividing the amount of the specific compound *n* *_i_* by the sum of the amounts of all considered products $n_{\text{products}}$, either all liquid products or all products, which might have been formed *via* Kolbe electrolysis.

(6) $$S_{i}=\frac{n_{i}}{\sum n_{\text{products}}}\times 100\%$$

The carbon balance was obtained by Equation (7) dividing the amount of carbon present in the quantified products, *n*_C,*i*_, by the amount of carbon contained in the consumed *n‐*CA, $\Delta n_{C,n-\mathrm{CA}}$. The amount of carbon *n*_C,*i*_ was calculated by multiplying the amount of molecules *n* *_i_* by the number of carbon atoms present in the molecule.

(7) $$\text{carbon balance}=\frac{\sum n_{C,i}}{\Delta n_{C,n-\mathrm{CA}}}\times 100\%$$

The acid degradation for single *n‐*CA or the total amount of *n‐*CA was calculated via Equation (8) by dividing the amount of the consumed *n‐*CA, $\Delta n_{n-\mathrm{CA}}$, by the amount of *n‐*CA present in the reaction solution at the start of the reaction $n_{n-\mathrm{CA},t_{0}}$.

(8) $$\text{acid degradation}=\frac{\Delta n_{n-\mathrm{CA}}}{n_{n-\mathrm{CA},t_{0}}}\times 100\%$$

### 4.7 Statistical Analysis

If not stated otherwise, all experiments were performed in at least three independent replicates (n≥3). In this context, independent replicates means that the preparation of the reaction solution, the electrolysis of the solution and the sample preparation for the GC–MS measurements were performed completely independently for every single replicate. All values are expressed as mean ± confidence interval (CI, α=0.05) unless otherwise specified.

## Conflicts of Interest

The authors declare no conflicts of interest.

## Supporting information

Supplementary Material

## Data Availability

The data that support the findings of this study are available from the corresponding author upon reasonable request.

## Endnotes

1 For N2, the data for the same electrode material as in the other works, platinized titanium Type B, was used.
