# Supplementary material for: The Concentration Determines Reaction Efficiency of Hetero‐Coupling of Bio‐Based Medium Chain Carboxylic Acids by Kolbe Electrolysis
Source: ChemSusChem. 2026 May 25;19(10):e70753. doi: 10.1002/cssc.70753 (PMC13206277; doi:10.1002/cssc.70753)
Supplement: Supplementary file 1 — Supplementary Material [file CSSC-19-e70753-s001.pdf]

Supporting Information

The Concentration Determines Reaction Efficiency of Hetero-Coupling of Bio-Based Medium Chain Carboxylic Acids by Kolbe Electrolysis

Max Pohl, Katharina Röhring, Micjel Chávez Morejón and Falk Harnisch*

Table of content of the SI

1. List of symbols and abbreviations 1
2. Kolbe electrolysis of n-CA 2
3. Schematic representation of electrochemical one-chamber configuration 3
4. Basic composition of reaction solution for Kolbe electrolysis 4
5. Electrochemical parameters 5
6. Classification of products from the Kolbe electrolysis 6
7. Chemicals 8
8. Gas phase analysis via microGC 9
9. Carbonate equilibrium 10
10. Overview of performance data for the different experiments 11
11. Yields of Kolbe and non-Kolbe products at different concentrations 12
12. Time-resolved oxygen evolution reaction for the long-term experiment 13
13. Acid degradation in long-term experiments 14
14. Calculation of the theoretical formation selectivity $\boldsymbol{S}_{\mathbf{theo,norm,i}}$ of *n*-alkanes from a *n*-CA mixture 15
15. Individual acid degradation for standard and long-term experiment 16
16. Calculation of the energy demand and electricity cost for fuel-production *via* Kolbe electrolysis 17

1. List of symbols and abbreviations

Abbreviations

CA	Carboxylic acid
CE	Counter electrode
CI	Confidence interval
CSR	Chain shortening reaction
DC	Direct current
DCM	Dichloromethane
DSP	Downstream processing
GC	Gas chromatography
HER	Hydrogen evolution reaction
MFM	Mass flowmeter
MS	Mass spectrometer
OER	Oxygen evolution reaction
RE	Reference electrode
TCD	Thermal conductivity detector
WE	Working electrode

Symbols

**Symbol**	**Unit**	**Meaning**
A	m^{-2}	Area
CE	%	Coulombic efficiency
E	V	Potential
F	A s mol^{-1}	Faraday constant (96 485 A s mol^{-1})
FE	-	Faraday equivalents
I	A	Electric current
M	kg mol^{-1}	Molar mass
P	-	*n*-octanol-water partition coefficient
P	W	Electrical power
Q	A s	Charge
S	%	Selectivity
T	°C	Temperature
V	L	Volume
W	J	Work
Y	%	Yield
c	mol L^{-1}	Concentration
n	mol	Amount of substance
n	-	Number of replicates
p	-	Probability
t	s	Time
x_i	-	Number of carboxylic acid molecules necessary to form the compound *i*
y	-	Mole fraction
z	-	Number of electrons transferred
α	-	Significance level
κ	$S^{-1}\ \mathrm{m}^{-1}$	Conductivity

2. Kolbe electrolysis of *n*-CA

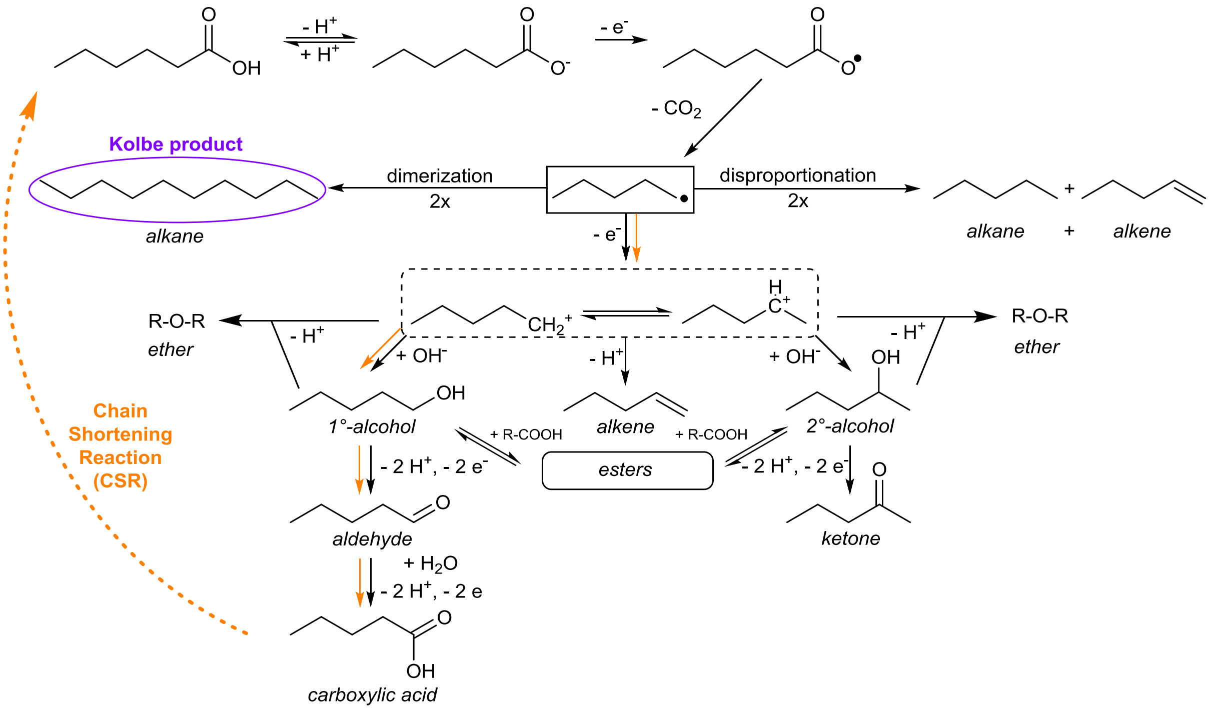

Figure S1. Reaction scheme of the mechanism of the Kolbe electrolysis for *n*-hexanoic acid (C_6) as exemplary *n*-CA, towards the Kolbe product (here: decane; purple circle) and its side products from disproportionation or further oxidation. The orange arrows show the chain shortening reaction (CSR) pathway leading to *n*-pentanoic acid (C_5).

3. Schematic representation of electrochemical one-chamber configuration

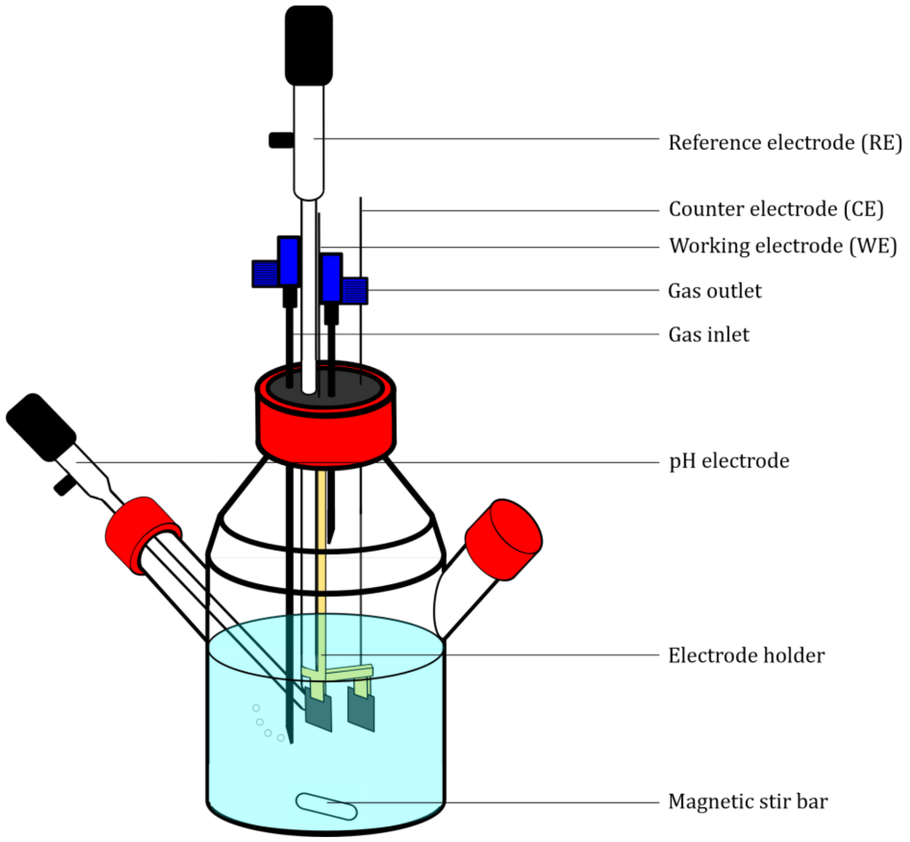

Figure S2. Schematic representation of electrochemical one-chamber configuration.

4. Basic composition of reaction solution for Kolbe electrolysis

Table S1. This overview shows the compounds of the basic composition of reaction solution for Kolbe electrolysis. For the *n*-CA, the proportion in the *n*-CA mixture and their maximal concentration in the 1.5 mol L^{-1} *n*-CA mixture is given. For the containing salt, Na_2CO_3, the concentration is constant independent of the *n*-CA solution concentration.

Compound	Proportion in *n*-CA mixture [%]	(max.) concentration [mmol L^{-1}]
acetic acid (C_2)	3.3	49.5
n-propanoic acid (C_3)	5.0	75.0
n-butanoic acid (C_4)	8.3	124.5
n-pentanoic acid (C_5)	3.3	49.5
n-hexanoic acid (C_6)	63.3	949.5
n-heptanoic acid (C_7)	10.0	150.0
n-octanoic acid (C_8)	6.7	100.5
Na_2CO_3	---	350.0

5. Electrochemical parameters

Table S2. This overview provides electrochemical parameters (Faraday equivalents (*FE*), current (*I*), total transferred charge (*Q*), time (*t*)) used to conduct the different short- and long-term experiments for the different starting *n*-CA concentrations $c(CA)_{t0}$. For the long-term experiment using 0 mol L^{-1} $c(CA)_{t0}$, the parameters were identical to that of the long-term experiment using 0.5 mol L^{-1} $c(CA)_{t0}$.

Short- or long-term experiment	$c(CA)_{t_0}$ [mol L^{-1}]	FE	I [mA]	Q [C]	t [min]
Short-term experiment	0.05	0.5	300	120.6	6.70
	0.10	0.5	300	241.2	13.40
	0.20	0.5	300	482.4	26.80
	0.30	0.5	300	723.6	40.20
	0.40	0.5	300	964.9	53.60
	0.50	0.5	300	1206.1	67.00
	1.00	0.5	300	2412.1	134.01
	1.50	0.5	300	3618.2	201.01
Long-term experiment	0.00	---	300	6030.3	335.02
	0.20	2.5	300	2412.1	134.01
	0.30	2.5	300	3618.2	201.01
	0.40	2.5	300	4824.3	268.01
	0.50	2.5	300	6030.3	335.02
	1.00	2.5	300	12060.6	670.03

6. Classification of products from the Kolbe electrolysis

Table S3. This overview summarizes the quantified compounds *i*, their assigned compound class and the number of electrons transferred to react or form this compound z_i in the *n*-CA mixture assuming a direct reaction pathway (possible CSR are not considered here; more details in section *"The impact of the chain-shortening reaction (CSR)"*). The compounds quantified by GC from the aqueous phase are marked with an a, those from the organic phase with b and those quantified by microGC with c. If the compounds were not calibrated and the quantification was done using an average response factor, they are further marked with *.

class	z_i	compound *i*	class	z_i	compound *i*
1° alcohol	2	1-butanol (ab)	*CO & CO_2*	*1*	CO (c)
		1-butanol, 2-ethyl- (b*)			CO_2 (c)
		1-heptanol (b)	*ester*	2	(E)-pent-2-en-3-yl acetate (b*)
		1-hexanol (ab)			(E)-pent-2-en-3-yl hexanoate (b*)
		1-nonanol (b*)			(Z)-pent-2-en-3-yl hexanoate (b*)
		1-octanol (b)			1-Phenylethyl hexanoate (b*)
		1-pentanol (ab)			2(3H)-Furanone, 5-ethyldihydro- (b*)
		1-pentanol, 2-methyl- (b*)			2-Pentanol, acetate- (b*)
		1-propanol (ab)			2-Propenoic acid, 2-methyl-, octyl ester (b*)
		4-penten-1-ol (b*)			2-Propenoic acid, butyl ester (b*)
		9-octadecen-1-ol (b*)			3-Heptyl hexanoate (b*)
		Ethanol (a)			Amyl heptanoate (b*)
2° alcohol	2	1-phenyl-1-decanol (b*)			Butanoic acid, 2-pentyl ester (b*)
		2-butanol (b)			Butanoic acid, 3,3-dimethyl-, methyl ester (b*)
		2-heptanol (b)			Butanoic acid, 3-heptyl ester (b*)
		2-hexanol (ab)			Butanoic acid, 3-pentyl ester (b*)
		2-pentanol (ab)			Butanoic acid, 6-ethyl-3-octyl ester (b*)
		2-propanol (ab)			Heptanoic acid, 2-methylpropyl ester (b*)
		3-heptanol (b)			Heptanoic acid, pentyl ester (b*)
		3-pentanol (ab)			Heptanoic acid, 3-methylbutyl ester (b*)
		4-decanol (b*)			Heptanoic acid, butyl ester (b*)
		5,6-decanediol (b*)			Hexanoic acid, 1-phenylethyl ester (b*)
		5-decanol (b*)			Heptanoic acid, heptyl ester (b*)
		5-nonanol (b*)			Heptyl caprylate (b*)
		5-undecanol, 2-methyl- (b*)			Hexanoic acid, 2-butyl ester (b*)
		5-undecanol (b*)			Hexanoic acid, 2-heptyl ester (b*)
aldehyde	4	Butanal (b*)			Hexanoic acid, 2-hexyl ester (b*)
		Butanal, 2-methyl- (b*)			Hexanoic acid, 2-methylbutyl ester (b*)
		Heptanal (b*)			Hexanoic acid, 2-pentyl ester (b*)
		Hexanal (b*)			Hexanoic acid, 2-phenylethyl ester (b*)
		Pentanal (b*)			Hexanoic acid, 2-propyl ester (b*)
aromatic compounds	-	Acetophenone (b*)			Hexanoic acid, 3-heptyl ester (b*)
		Benzaldehyde (b*)			Hexanoic acid, 3-pentyl ester (b*)
	2	Benzene, heptyl- (b*)			Hexanoic acid, heptyl ester (b*)
	-	Benzene, hexyl- (b*)			Hexanoic acid, hexyl ester (b*)
	2	Benzene, nonyl- (b*)			Hexanoic acid, octyl ester (b*)
		Benzene, octyl- (b*)			Hexanoic acid, pentyl ester (b*)
	-	Benzene, pentyl- (b*)			Hexanoic acid, phenylmethyl ester (b*)
		Benzenemethanol, .alpha.-methyl- (b*)			Octan-2-yl 2-methylbutanoate (b*)
		Ethylbenzene (b*)			Octanoic acid, 2-butyl ester (b*)
		Heptanophenone (b*)			Octanoic acid, 2-butyl ester (b*)
		Phenylethyl Alcohol (b*)			Octanoic acid, 2-heptyl ester (b*)
carboxylic acid	2	5-Methylhexanoic acid (b*)			Octanoic acid, 2-hexyl ester (b*)
		Acetic acid (a)			Octanoic acid, 2-pentyl ester (b*)
		Benzeneacetic acid (b*)			Octanoic acid, 2-propyl ester (b*)
		Benzoic acid (b*)			Octanoic acid, 3-heptyl ester (b*)
		Benzyl alcohol (b*)			Octanoic acid, 3-pentyl ester (b*)
		Butanoic acid (ab)			Octanoic acid, heptyl ester (b*)
		Butanoic acid, 2-methyl- (b*)			Octanoic acid, pentyl ester (b*)
		Butanoic acid, 3-methyl- (a)			Pentanoic acid, 2-pentyl ester (b*)
		Decanoic acid (b)			Pentanoic acid, 3-pentyl ester (b*)
		Cyclohexanecarboxylic acid (b*)			Pentanoic acid, pentyl ester (b*)
		Heptanoic acid (ab)	*Gaseous hydrocarbons*	2	Butane + Butene (c)
		Hexanoic acid (ab)			Methane (c)
		Nonanoic acid (b)			Ethane + Ethylene (c)
		Hydrocinnamic acid (b*)			Hexane (c)
		Octanoic acid (ab)			Pentane + Pentene (c)
		Pentanoic acid (ab)			Propane + Propene (c)
		Propanoic acid (a)	*H_2*	2	H_2 (c)
		Propanoic acid, 2-methyl- (a)			
		Pentanoic acid, 4-methyl- (b*)			

class	z_i	compound *i*	class	z_i	compound *i*
n-alkane[1]	1	Butane (b*)	*other*	4	Dipentylether (b*)
	2	Decane (b)		-	Carbonic acid, dipentyl ester (b*)
		Dodecane (b)			Enanthamide (b*)
		Heptadecane (b)			Fumaric acid, decyl 2-phenylethyl ester (b*)
	1	Heptane (b) [2]			Fumaric acid, nonyl 2-phenylethyl ester (b*)
	2	Hexadecane (b)			Furan, tetrahydro-2,5-dimethyl- (b*)
		Hexane (b)			Heptanoic acid, anhydride (b*)
		Nonane (b)			n-Amyl ether (b*)
		Octadecane (b)			Oxalic acid, isobutyl pentyl ester (b*)
		Octane (b)			Pentane, 2,2'-oxybis- (b*)
		Pentadecane (b)			Propane, 2-ethoxy-2-methyl- (b*)
	1	Pentane (b) [2]	*Other hydrocarbons*	1	3-Heptene, (Z)- (b*)
	2	Tetradecane (b)			1-Heptene (b*)
		Tridecane (b)			1-Hexene (b*)
		Undecane (b)			1-Pentene (b*)
O_2	4	O_2 (c)			2-Heptene, (E)- (b*)
				2	Decane, 2-methyl- (b*)
					Heptane, 3-ethyl- (b*)
					Nonane, 5-methyl- (b*)
					Octane, 2-methyl- (b*)
					Undecane, 2,3-dimethyl- (b*)

[1] It is assumed that the pathway for the *n*-alkane production in this *n*-CA mixture is the hetero- and homo coupling of different radicals leading to z_{alkane} = 2. Exception: *n*-heptane and *n*-pentane (compare to [2]).
[2] The 2-electron-pathway to produce *n*-heptane and *n*-pentane is a hetero-coupling of alkyl radicals from *n*-propanoic, *n*-butanoic and/or *n*-pentanoic acid. The 1-electron-pathway is the disproportionation of *n*-hexanoic or *n*-octanoic acid. Due to the higher concentration of *n*-hexanoic and *n*-octanoic acid in the *n*-CA mixture, it is assumed that the favored pathway is the 1-electron-pathway resulting in $z_{n-\mathrm{pentane}\ /n-\mathrm{heptane}}$ = 1.

7. Chemicals

2-hexanol (98%) and 3-heptanol (98%) were supplied by abcr GmbH (Karlsruhe, Germany). Sodium carbonate (≥ 99%, anhydrous) and sulfuric acid (98%) were purchased by Carl Roth GmbH + Co. KG (Karlsruhe, Germany). Acetic acid (> 99%) and isopentanoic acid (98%) were obtained from Fluka Chemie GmbH (Buchs, Switzerland). Dichloromethane, *n*-hexane and sodium hydroxide were supplied by Merck KGaA (Darmstadt, Germany). 1-butanol (99%, analytical standard), 1-pentanol (99%, analytical standard), 1-propanol (99%, analytical standard), cyclohexanone (99%, analytical standard), isobutyl hexanoate (98%), *n*-butanoic acid (≥ 99%), *n*-decane (99%, analytical standard), *n*-dodecane (99%, analytical standard), *n*-heptadecane (99%, analytical standard), *n*-heptanoic acid (≥ 99%), *n*-hexanoic acid (≥ 98%), *n*-nonane (99%, analytical standard), *n*-octane (99%, analytical standard), *n*-octanoic acid (≥ 99%), *n*-pentadecane (99%, analytical standard), *n*-pentanoic acid (≥ 99%), *n*-propanoic acid (≥ 99.5%), *n*-tetradecane (99%, analytical standard), *n*-tridecane (99%, analytical standard), *n*-undecane (99%, analytical standard), undecanoic acid (99%) and undecanoic acid methyl ester (analytical standard) were purchased by Sigma-Aldrich Chemie GmbH (Steinheim, Germany). The carrier gases helium and argon (99.999 vol% purity) and the calibration gases for level 1, 2, 5 and 6 (composition see Table S5) for gas chromatography as well as N_2 (> 99vol%) were obtained from Air Products GmbH (Hattingen, Germany). The calibration gas for level 3 and 4 were supplied by Air Liquid S.A. (Paris, France) and by Praxair N.V. (Oevel, Belgium).

8. Gas phase analysis via microGC

Table S4. Specification of the microGC-TCD used and method for gas composition analysis.

Column/ Parameter	Unit	RT-Molsieve 5A (0.25 mm, 10 m) with Rt-Q-BOND pre-column (3 m), 1 µL backflush injector	RT-Q-Bond (0.25 mm, 12 m), fixed volume injector
Carrier gas		Argon	Helium
Sample inlet temperature	°C	100	100
Injector temperature	°C	90	90
Column temperature profile		80 °C	60 °C for 30 s<br>1 K s^{-1} for 170 s<br>230 °C for 30 s
Injection time	ms	0	30
Column pressure	psi	25	20
Analyzed components		H_2, O_2, N_2, CO, CH_4	CO_2, CH_4, Ethane + Ethylene, Propane + Propylene, Butane + Butene, Pentane + Pentene, *n*-hexane

Table S5. Components and concentrations in the different calibration gases used for the calibration of the microGC-TCD.

Component	Level 1	Level 2	Level 3	Level 4	Level 5	Level 6
1,3-butadiene			0.3			
1-butene	5	0.1	0.3			
1-pentene			0.1			
2-*cis*-butene			0.3			
2-*cis*-pentene			0.05			
2-methyl-2-butene			0.05			
2-*trans*-Butene			0.15			
2-trans-Pentene			0.05			
acetylene			1			
argon			0.19			
CO			1	10		
CO_2			3	20		
ethane	0.1	5	4			
ethylene	5	0.1	2			
H_2	0.1	5	10			99.999
iso-butane			0.3			
iso-butene			0.15			
iso-pentane			0.1			
methane	5	0.1	5			
N_2	79	78.5	Rest	70	78.1	
n-butane	0.1	5	0.15			
n-heptane			0.05			
n-hexane	0.5	0.1	0.05			
n-pentane	0.1	1	0.05			
O_2					20.9	
propane	0.1	5	2		1	
propylene	5	0.1	1			

9. Carbonate equilibrium

$$CO_2 + H_2O \leftrightarrows H_2CO_3 \qquad \text{Eq. S 1}$$

$$H_2CO_3 + OH^- \leftrightarrows HCO_3^- + H_2O \qquad pK_\mathrm{a} = 6.36^{[32]} \qquad \text{Eq. S 2}$$

$$HCO_3^- + OH^- \leftrightarrows CO_3^{2-} + H_2O \qquad pK_\mathrm{a} = 10.34^{[32]} \qquad \text{Eq. S 3}$$

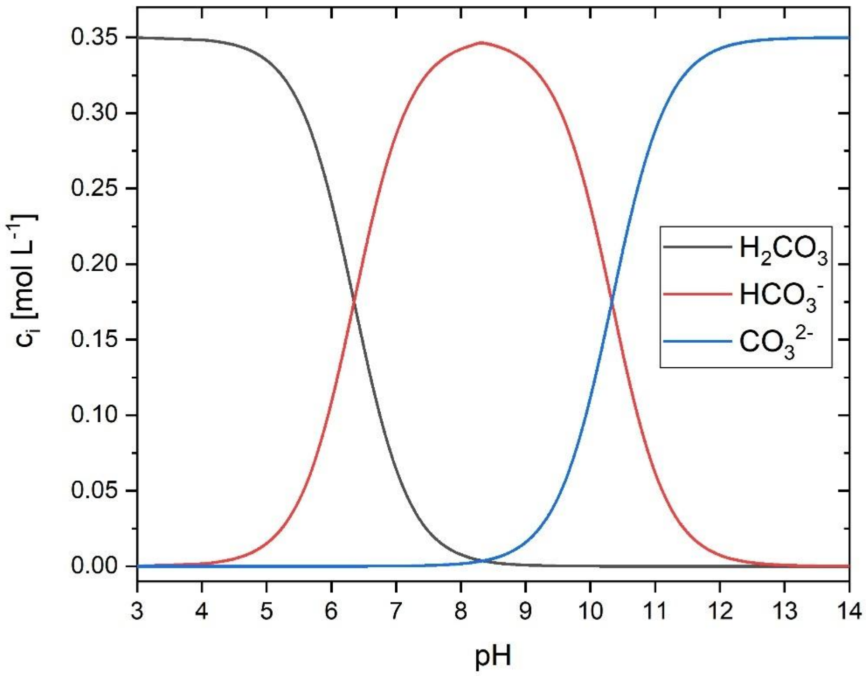

Figure S3. Concentration of different carbonate species (H_2CO_3, HCO_3^- and CO_3^{2-}) depending on the pH of the solution. 0.35 mol L^{-1} is assumed as initial carbonate concentration. The ratios of H_2CO_3 to HCO_3^- and of HCO_3^- to CO_3^{2-} were calculated using the Henderson-Hasselbalch equation $\left(\frac{c(A^-)}{c(HA)} = 10^{pH-pK_\mathrm{a}}\right)$. The concentrations of the single species were calculated using the ratio of H_2CO_3 to HCO_3^- up to pH = 8.3. From there on, the calculation used the ratio of HCO_3^- to CO_3^{2-}, assuming that the concentration of the third species is negligible within those pH ranges.

10. Overview of performance data for the different experiments

Table S6. Yield (Y_{fuel}), selectivity (S_{fuel}), CE_{fuel} for the sum of fuel-like liquid products in the liquid phase per converted *n*-CA; acid degradation; as well as starting pH pH_{start} and conductivity κ_{start} for the standard experiments (0.5 FE) and long-term experiments (2.5 FE) for initial concentrations ($c(CA)_{t_0}$) ranging from 0.05 mol L^{-1} up to 1.5 mol L^{-1}. CE_{overall} includes gaseous hydrocarbons, not CE_{OER}. $CE_{\text{liquid hydrocarbons}}$ include all hydrocarbons quantified in the liquid, organic phase. $CE_{\text{non-Kolbe}}$ include all alcohols, esters and aldehydes quantified in the liquid phase. CE_{OER} and CE_{H_2} are compound-specific CE for evolution of O_2 and H_2, respectively. The number of replicates for the short-term experiment at 0.5 mol L^{-1} is n = 5 and for the gaseous compound related parameters for standard experiment at 0.4 mol L^{-1} and for all long-term experiments is n = 1.

Short- or long-term experiment	$c(CA)_{t_0}$ [mol L^{-1}]	Y_{fuel} [%]	S_{fuel} [%]	CE_{fuel} [%]	CE_{anode} [%]	CE_{overall} [%]	$CE_{\text{liquid hydrocarbons}}$ [%]	$CE_{\text{non-Kolbe}}$ [%]	CE_{OER} [%]	CE_{H_2} [%]	pH_{start}	κ_{start} [mS cm^{-1}]	*Acid degradation* [%]
Short-term experiment (0.5 FE)	0.03±0.00	7.4±3.5	8.4±5.6	0.9±0.2	92.9±21.8	6.0±2.7	0.0±0.0	0.9±0.2	86.9±21.1	86.2±11.5	9.1±0.1	43.1±1.0	0.9±10.3
	0.09±0.00	9.5±11.9	40.5±4.5	2.8±1.2	68.1±43.9	4.8±2.4	0.1±0.2	2.7±1.1	63.4±45.5	81.0±16.6	9.1±0.0	44.2±0.9	-9.1±6.9
	0.19±0.01	20.0±13.6	73.2±10.3	10.5±1.9	42.5±27.6	12.6±2.9	2.6±0.9	7.9±1.4	29.9±30.5	83.3±15.1	9.1±0.0	46.5±1.4	19.2±13.5
	0.28±0.01	48.5±21.1	69.4±11.1	12.4±6.3	33.8±4.5	15.3±8.1	6.0±4.4	6.4±2.3	18.5±11.0	86.4±16.6	9.0±0.0	48.1±0.6	10.6±2.8
	0.37±0.03	87.9±94.9	84.9	23.9±2.2	41.0	26.7	16.9±2.7	7.0±2.1	14.3±0.0	112.0±0.0	9.0±0.1	50.1±0.6	16.5±20.3
	0.46±0.05	47.3±10.3	86.7±10.6	32.2±6.0	48.5±9.1	35.3±7.9	25.4±5.8	6.8±1.3	13.2±7.3	120.4±30.4	9.1±0.1	50.8±1.3	34.3±2.5
	0.98±0.03	60.5±7.6	82.5±2.4	46.5±8.7	58.8±5.4	51.8±8.9	40.9±8.3	5.6±0.8	7.0±4.3	104.2±5.2	9.0±0.0	57.6±4.3	37.8±5.8
	1.51±0.08	83.3±16.5	85.3±0.9	63.8±1.4	73.4±2.1	69.6±1.1	56.5±2.0	7.3±1.0	3.9±1.2	105.7±6.5	9.1±0.1	58.9±1.1	37.3±4.9
Long-term experiment (2.5 FE)	0.18	25.7	61.5	8.5	31.7	11.3	2.5	5.9	20.4	97.9	9.3	49.4	61.1
	0.30	33.7	64.5	13.0	37.0	16.7	4.6	8.4	20.3	95.4	9.2	46.9	66.9
	0.39	41.6	60.4	16.2	37.1	21.7	8.0	8.2	15.4	94.5	9.2	47.7	76.9
	0.45	45.6	56.0	16.2	40.8	23.1	9.5	6.7	17.8	95.8	9.1	54.7	83.2
	1.00	56.0	62.9	22.5	46.8	29.4	16.3	6.3	17.4	95.0	9.4	52.4	89.2

11. Yields of Kolbe and non-Kolbe products at different concentrations

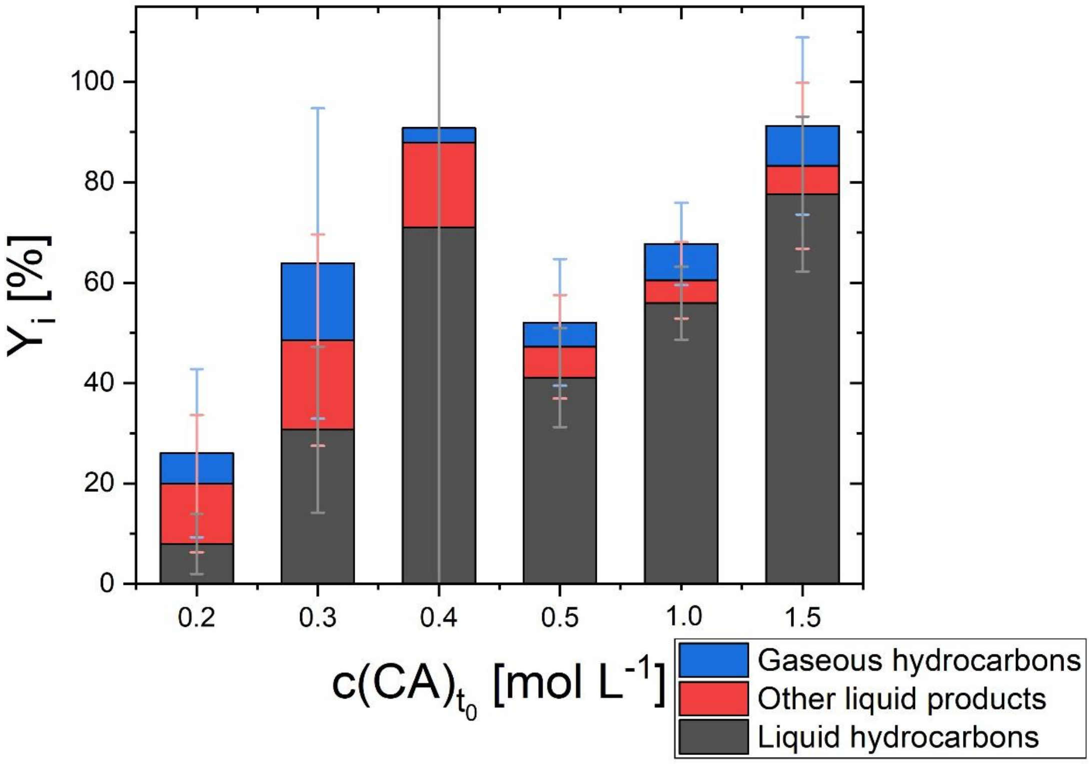

Figure S4. Yields Y_i of liquid hydrocarbons, other liquid products and gaseous hydrocarbons relative to the degraded *n*-CA for the different initial *n*-CA concentrations ($c(CA)_{t_0}$) in a range from 0.2 mol L^{-1} to 1.5 mol L^{-1}.

12. Time-resolved oxygen evolution reaction for the long-term experiment

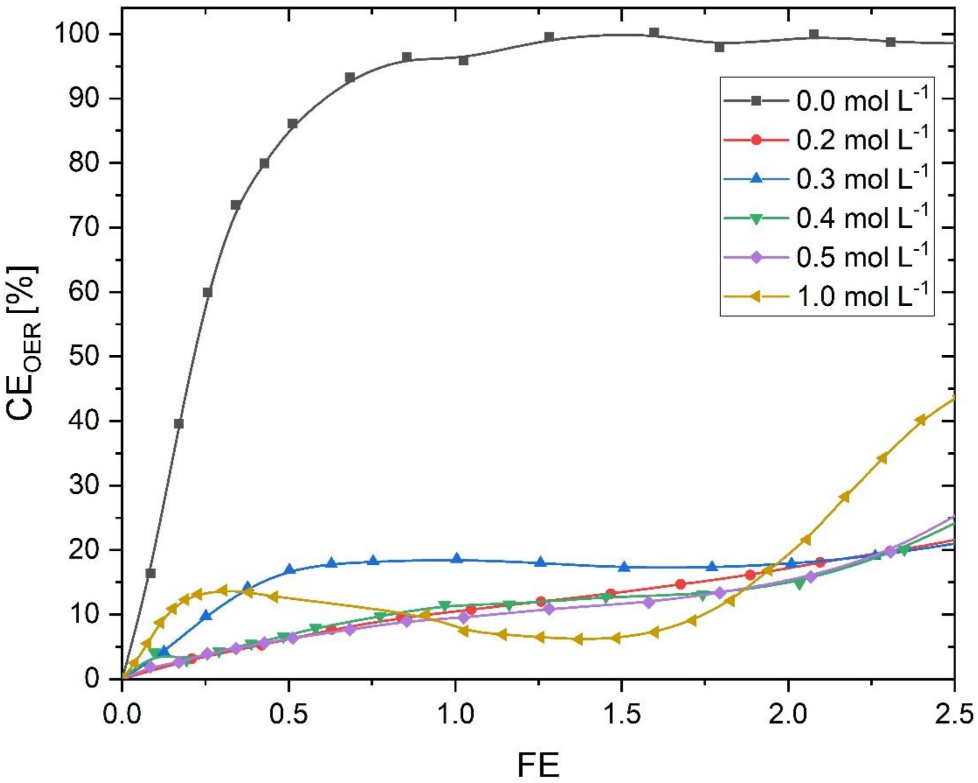

Figure S5. Plot of CE_{OER} for 2.5 FE in the long-term experiments for concentrations of the *n*-CA mixture between 0 mol L^{-1} and 1.0 mol L^{-1}. FE gives a relative time scale in dependency of the transferred charges relative to the starting concentration. FE of 0 mol L^{-1} is normalized to the transferred charges of the experiment using 0.5 mol L^{-1}. For 0.5 mol L^{-1}, C_8 is missing in the *n*-CA mixture.

13. Acid degradation in long-term experiments

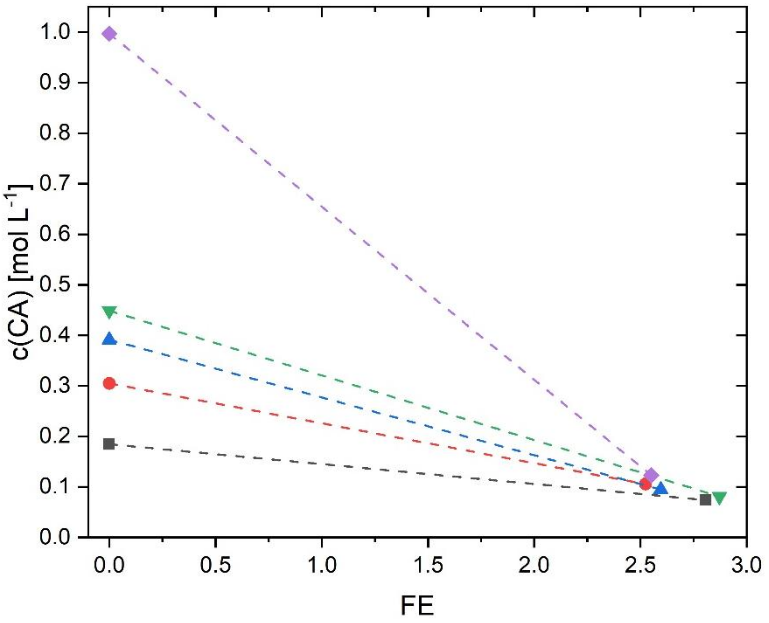

Figure S6. Concentration of carboxylic acids $c(CA)$ relative to the transferred charges FE for the long-term experiment for different initial concentration in a range of 0.2 mol L^{-1} to 1.0 mol L^{-1}.

14. Calculation of the theoretical formation selectivity $S_{\mathrm{theo,norm,i}}$ of *n*-alkanes from a *n*-CA mixture

Example for the calculation:

From the *n*-CA mixture the same product can be formed by different reactions pathways, as exemplified for *n*-dodecane. It can be gained by coupling reaction of radicals formed via Kolbe electrolysis from C_6 (i.e. C_5●) with C_8 (i.e. C_7●), C_8 (i.e. C_7●) with C_6 (i.e. C_5●) or C_7 with C_7 (i.e. 2 × C_6●). Based on the proportion in the *n*-CA mixture (see Table S1), an encounter probability can be calculated by multiplying both shares (C_6 = 63.3%; C_7 = 10.0%; C_8 = 6.7%). This leads to the encounter probabilities p of $p_{\mathrm{C_6+C_8}} = 4.2\%$; $p_{\mathrm{C_8+C_6}} = 4.2\%$ and $p_{\mathrm{C_7+C_7}} = 1.0\%$ for coupling of the respective two radicals to form *n*-dodecane. Summing up, these probabilities results in the total coupling probability or theoretical selectivity for *n*-dodecane $S_{\mathrm{theo},n-\mathrm{dodecane}} = 9.4\%$. This theoretical selectivity for *n*-dodecane is normalized to all *n*-alkanes with eight or more C atoms, which cannot be formed *via* disproportionation. This results in $S_{\mathrm{theo,norm},n-\mathrm{dodecane}} = 11.1\%$.

Table S7. Overview of the data for the calculation of the theoretical formation selectivity of *n*-alkanes from a *n*-CA mixture. The *n*-alkane chain length i, their theoretical formation selectivity $S_{theo,i}$ and the theoretical, normalized selectivity for *n*-alkanes $S_{theo,norm,i}$with eight or more C atoms are given as results of the calculation. Further, the normalized selectivity $S_{norm,i}$ for the experiments using 0.5 FE and initial concentrations $(c(CA))_{t_0}$ ranging from 0.5 mol L^{-1} to 1.5 mol L^{-1} are given. The number of replicates at 0.5 mol L^{-1} is n = 5.

***n*-alkane chain length i of Kolbe product**	$S_{\mathrm{theo,i}}$ **[%]**	$S_{\mathrm{theo,norm},i}$ **[%]**	$S_{\mathrm{norm,i}}$ **[%]** $c = 0.5\ \mathrm{mol\ L^{-1}}$	$S_{\mathrm{norm,i}}$ **[%]** $c = 1.0\ \mathrm{mol\ L^{-1}}$	$S_{\mathrm{norm,i}}$ **[%]** $c = 1.5\ \mathrm{mol\ L^{-1}}$
2	0.1	---	---	---	---
3	0.3	---	---	---	---
4	0.8	---	---	---	---
5	1.1	---	---	---	---
6	5.3	---	---	---	---
7	7.6	---	---	---	---
8	12.1	14.3	2.8±0.6	2.3±0.1	2.1±0.2
9	6.6	7.7	3.3±0.6	2.8±0.1	2.8±0.1
10	41.9	49.3	41.9±7.0	48.1±2.9	47.1±0.2
11	13.1	15.4	23.9±1.9	22.2±0.8	22.7±0.4
12	9.4	11.1	20.4±1.1	18.9±1.0	20±0.2
13	1.3	1.6	3.2±1.7	4.1±0.3	3.9±0.1
14	0.4	0.5	4.6±7.7	1.4±0.2	1.5±0.4

15. Individual acid degradation for standard and long-term experiment

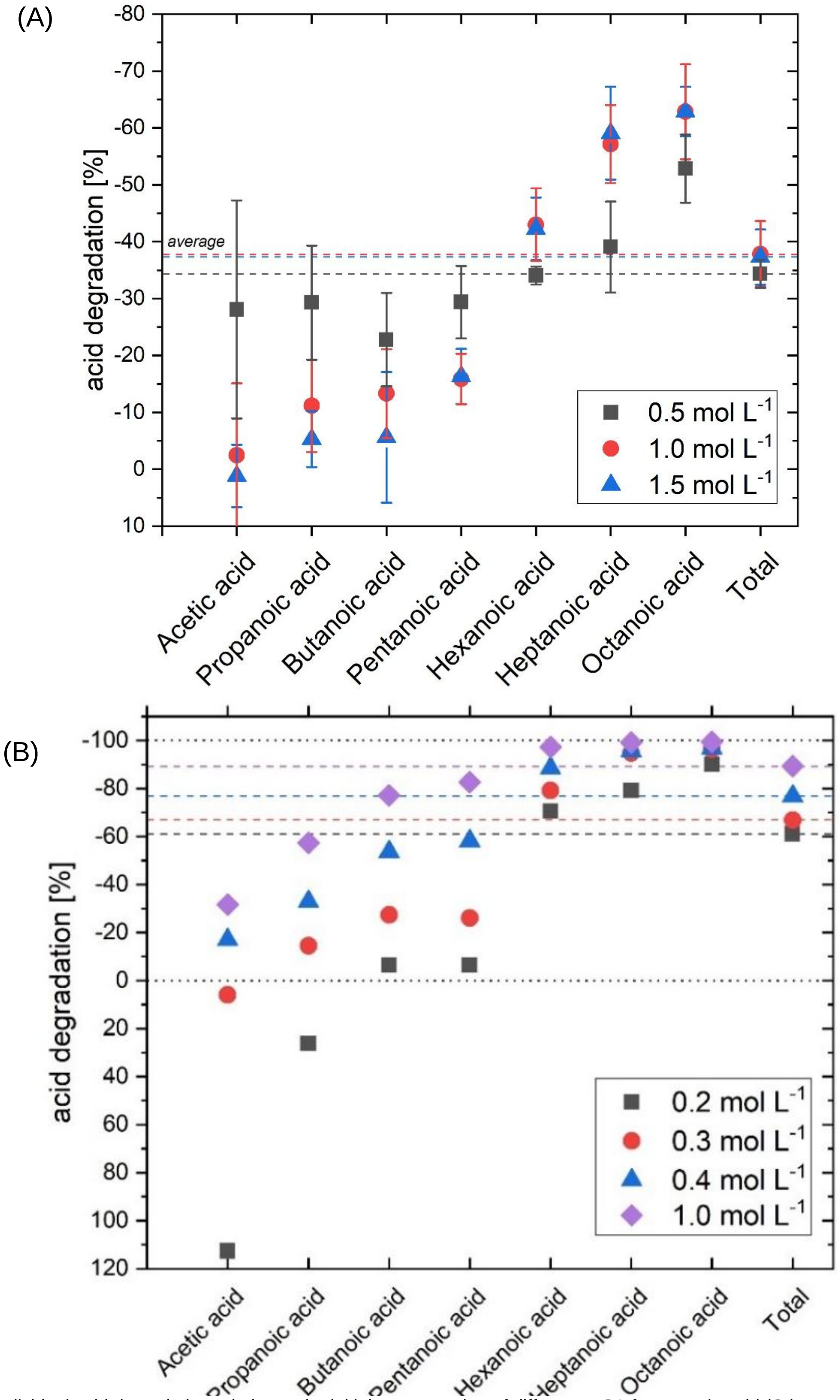

Figure S7. (A) Individual acid degradation relative to the initial concentration of different *n*-CA from acetic acid (C_2) to octanoic acid (C_8) and the average acid degradation, which is marked with a dashed line, for different initial *n*-CA concentrations of 0.5 mol L^{-1} to 1.5 mol L^{-1} for the standard experiment (0.5 FE). (B) Individual acid degradation relative to the initial concentration of different *n*-CA from acetic acid (C_2) to octanoic acid (C_8) and the average acid degradation, which is marked with a dashed line, for different initial *n*-CA concentrations of 0.2 mol L^{-1} to 1.0 mol L^{-1} in the long-term experiment (2.5 FE). Positive values indicate the formation of *n*-CA, while negative values indicate the degradation and hence conversion to products.

16. Calculation of the energy demand and electricity cost for fuel-production *via* Kolbe electrolysis

This calculation is adopted from NEUBERT *et al.*[15]

The electrical power, P_{el}, is gained from the cell voltage, E_{cell}, and the current, I (Eq. S 4). Since the electrolysis was run in galvanostatic mode, E_{cell} fluctuated throughout the duration of the experiment. Therefore, P_{el}, was calculated for each measurement point individually over the duration of the electrolysis. With Eq. S 5 the electric energy input, W_{cell}, needed for the Kolbe electrolysis is calculated by integrating the electrical power, P_{el}, over the duration of the experiment, t. To facilitate energy input comparison, the consumed energy was related to n_{fuel}.

$$P_{\text{el}} = E_{\text{cell}} \cdot I \qquad \text{Eq. S 4}$$

$$W_{\text{cell}} = \int P_{\text{el}}(t)dt \qquad \text{Eq. S 5}$$

The parameters for the performance of the Kolbe electrolysis of an *n*-CA mixture, *i.e. n*-CA concentration, E_{cell}, j, CE_{fuel} and fuel production rate are based on lab scale data.

Table S8. Parameters based on the electrolysis of the *n*-CA mixture with an initial *n*-CA concentration of 1.5 mol L^{-1}, presented in this paper for the calculation of fuel costs. Presented are average values ± 95% confidence interval.

**Parameter**	**Unit**	
n-CA concentration (total)	mol L^{-1}	1.51±0.08
Electrolysis time	min	201.1
Current I	A	0.3
Produced fuel	mmol	11.50±0.26
E_{cell} (start and end value)	V	7.19±0.54 – 6.51±0.22
W_{cell}	kWh	6.68±0.23
Energy consumption per fuel	kWh mol^{-1}	0.58±0.02
Commercial electricity price Germany (Industry)[33]	€ct kWh^{-1}	17.8

As it can be seen in Table S8, the Kolbe electrolysis of an *n*-CA mixture with an initial concentration of 1.51±0.08 mol L^{-1} done for 201.1 min (0.5 FE) consumed 0.58±0.02 kWh per mole of fuel produced. Fuel in this regard includes all products present in the liquid organic phase since all formed compounds have fuel properties. The electrolysis yielded 11.50±0.26 mmol fuel. That is about 2.28±0.05 mL fuel ($\text{Ø}MW$ = 148.6 ± 3.0 g mol^{-1}, $\text{Ø}\rho$ = 0.741±0.004 g mL^{-1}, calculated from the shares of all considered products) per batch experiment. This leads to an estimate that 1 L fuel can be produced using 2.92±0.04 kWh electric energy. This results in an electricity price of 52.0±0.7 €ct per liter of fuel.
